# A Descriptive Study of the Attitudes, Characteristics and Behaviours Differentiating Men Who Do and Do Not Want Help for Their Sexual Interest in Children

**DOI:** 10.1177/08862605251403618

**Published:** 2026-03-03

**Authors:** Michael Salter, Tyson Whitten, Delanie Woodlock, Mengyao Lu, Maria Lamond, Georgia Naldrett, Matt Tyler

**Affiliations:** 1 University of New South Wales, Sydney, Australia; 2 University of Edinburgh, Scotland; 3 Jesuit Social Services, Richmond, VIC, Australia

**Keywords:** offenders/perpetrators, child maltreatment, prevention, child maltreatment, internet and abuse, technology- or image-based abuse, violence prevention

## Abstract

In the field of child sexual abuse prevention, secondary prevention services seek to engage with people concerned about their sexual interest and behaviours towards children prior to the onset of offending. There is a lack of robust information to inform program development and to seek to engage earlier with people concerned about their risk to children. Accordingly, this paper reports on the findings of a survey of 4,918 men, drawn from representative samples in Australia, the United Kingdom, and the United States. The paper described the proportion of men in the survey with a sexual interest in children (n = 642), and the factors that distinguish between men who wanted help for that interest and men who did not want help. Study findings indicate that offending behaviour is a key motivator for help-seeking amongst men with a sexual interest in children. Men who wanted help were significantly more likely to be married or living with a partner and working with children, and they reported closer social bonds, compared to men with sexual feelings who did not want help, which suggests that their help-seeking may be motivated by concern about the impact of their offending on their close relationships, social ties, and reputation. This group also displayed heightened rates of other forms of sexual deviancy, such as arousal to animal pornography, as well as the use of encrypted social media apps, potentially to camouflage or hide their offending activity. These findings may be useful for secondary prevention services in order to build motivation to seek help amongst men with a sexual interest in children.

## Introduction

State responses to child sexual abuse have focused on the post-offence phase, including punishment and deterrence via the criminal justice system, and the rehabilitation of perpetrators and victims. Child sexual abuse prevention efforts have been evident for at least 40 years and include education programs aimed at children and parents, child safeguarding provisions in institutional settings (Quadara et al., 2015), and the criminalisation of child sexual abuse material (CSAM) and other products, such as child sexual abuse dolls, that encourage or normalise offending (Brown & Shelling, 2019). “Safety by design” provisions that aim to embed child protection into software design are a more recent prevention development (Finkelhor et al., 2022). Expanding interest in a coordinated, public health approach to child sexual abuse has focused attention on opportunities to engage early with people who pose a risk to children, with the aim of preventing offending or reoffending. Such services have been active in some jurisdictions for at least 30 years and experience a high level of demand (De Boeck et al., 2022). While these services aim to engage with clients prior to the onset of offending, a significant proportion of clients disclose criminal offences against children that have not been detected by the authorities (De Boeck et al., 2022). However, there is a lack of robust information to inform program development and efforts to intervene earlier in offending pathways, such as the proportion and characteristics of people in the community who are more likely than others to commit a child sex offence. Based on the findings of a survey of 4,918 men, drawn from the general Australian, U.K., and U.S. adult male populations, this paper describes the proportion of men with a sexual interest in children, and the factors that distinguish between men who wanted help for that interest and those who do not. The paper closes by reflecting on the implications of these findings for early intervention and secondary prevention of child sexual abuse.

## Literature Review

A recent systematic review of research into the prevalence of sexual interest in children reported a prevalence range of 2% to 24%, with the degree of variance linked to inconsistent definitions and measures as well as different methodological approaches (Savoie et al., 2021). Such studies suggest that there is a significant cohort of people in the community with a sexual interest in children, only some of whom have acted on that interest, and even less of those have come to the attention of the authorities.^1^ Psychological treatment and support for people with a sexual interest in children is mostly concentrated in the criminal justice system, targeting people convicted of a sexual offence. People who are concerned about their sexual interest or behaviours towards children, but have not acted or have not been caught, are generally unable to access specialist psychological treatment due to lack of available services and fear of stigma and criminal consequences. In the absence of mental health support, men with a sexual interest in children evince a range of harmful coping strategies and mental health outcomes such as anxiety, depression, addiction, self-hatred, and suicide ideation (Stevens & Wood, 2019). However, negative emotional states such as depression and stress have been linked to offending amongst men who commit online offences against children (Wortley et al., 2024).

Scholars with an interest in promoting help-seeking amongst people with a sexual interest in children have focused, in particular, on the barriers of stigma and shame (Kewley et al. 2023). Like other deviant or harmful impulses, sexual interest in children is a stigmatised experience that paedophiles or hebephiles may seek to keep secret from those around them, although some will seek out online communities of like-minded individuals (Goode, 2009). Accordingly, some scholars claim that the stigma of sexual interest in children should be challenged or reduced to encourage such individuals to seek treatment (Lawrence & Willis, 2021). Elsewhere, we have questioned this rationale on several grounds, including that stigma is not synonymous with discrimination, and serves to delineate socially acceptable from socially unacceptable behaviour (Farmer et al., 2024). Calls for the normalisation of sexual interest in children are a feature of pro-paedophile activism and serve as the cultural foundation of online communities of child sex offenders (Goode, 2009; Huikuri, 2023), which further challenges the proposition that the normalisation project is a preventative one. Nonetheless, paedophiles who have approached mental health providers and disclosed sexual interest in children have reported discrimination and a lack of preparedness in clinical settings (Grady et al., 2019; Levenson & Grady, 2019). A lack of professional training and guidance for psychologists, as well as uncertainty about the legal obligations of treatment providers working the community with clients disclosing a sexual interest, seriously restricts the availability of mental health care to paedophiles and other people with a sexual interest in children (Austin & Salter, 2024).

There are now several services and programs that aim to address this gap, and support people who are concerned about their sexual interest or behaviours to abstain from offending. Of these initiatives, Stop it Now! and the Prevention Project Dunkelfeld (PPD) are the most well-known (Knack et al., 2019). The scale of demand for both programs demonstrates the existence of a significant minority of people in the community who are looking for support to prevent or abstain from sexually harming children, but evaluations of both programs also point to the challenge of connecting early with such individuals to prevent offending onset. Stop it Now! originated in the United States in 1992 and has since expanded to include affiliate sites in the United Kingdom and Ireland, Wales, the Netherlands, Scotland, Belgium (De Boeck et al., 2022) and, recently, Australia. While the exact model of service varies from country to country, the cornerstone of Stop It Now! is an anonymous helpline that offers support for people to manage sexual interest or behaviours towards children, and to provide advice to people concerned about the risk posed by someone in their family, workplace or other settings. In the United Kingdom, between half to four fifths of individuals seeking help for themselves who contact the Stop It Now! helpline report that they have already engaged in child sexual offending of some form (B. J. Grant et al., 2019; Horn et al., 2015). While Stop It Now! services show strong demand, evidence of preventative effect has been difficult to gather due to the anonymous nature of the service (De Boeck et al., 2022).

The PPD began as a research project in Berlin in 2005 and has evolved into the Prevention Network, with treatment providers situated across Germany (Beier et al., 2015). Whereas the Stop It Now! model hinges on an anonymous hotline, PPD provides in-person treatment to people with a sexual interest in the community, who may or may not be known to authorities. PPD operates in a legislative environment in which treatment providers do not have a mandatory obligation to report a client to the authorities who is currently abusing a child or intends to abuse a child (Beier et al., 2021). As a result, PPD can gather more comprehensive data on offending history and response to treatment than services under proactive reporting obligations. While an evaluation of clients who completed the program reported some reduction in dynamic risk factors, self-esteem also worsened amongst the client group, there was no reduction in offending behaviour, and one quarter of clients with no history of CSAM offending began offending (Beier et al., 2015).

A key challenge for secondary prevention programs is that they seek to engage cohorts who may be distinct in their underlying criminogenic risk and needs, including pre-offenders (i.e. those who evince sexual interest or problematic behaviours towards children but have not yet offended), undetected offenders (i.e. those who have or are offending but are not currently known to law enforcement), and offenders who have been arrested and are known to the authorities. The nature of the offending behaviours disclosed by clients of secondary prevention services can vary, and these different groups may require different forms of intervention (Stephens et al., 2022). However, relatively little is known about why some men want help for their sexual interest or behaviours towards children, why some do not, and what factors distinguish between them. A consistent finding of secondary prevention services is that most clients who make contact to express concerns about their own interest or behaviours have already offended against children, which has prompted services to consider how they might engage earlier. For instance, in September 2023, Stop It Now! UK launched a bespoke website for teenagers aged 13 to 18 who are concerned about their sexual behaviours, including viewing CSAM (H. Grant, 2023). Other than targeting adolescents, it is unclear how people with a sexual interest or concerning behaviours towards children might be encouraged to seek help earlier in the offending pathway, or how secondary prevention services might expand their engagement with at risk populations. The aim of this paper is to analyse the differences between men with sexual interest in children who do and do not want help for that interest, with the aim of identifying opportunities to connect more effectively and earlier with this group of men in order to prevent child sexual abuse before it occurs.

## Methods

### Data

An online survey was conducted examining the prevalence and factors associated with men’s sexual attitudes, interests, and behaviours towards children between November and December 2022. The survey was developed with a focus on public policy responses and prevention opportunities, and hence asked a wide range of questions about men’s background, health, social context, internet use, and online activities, in order to develop a detailed picture of the circumstances of participants, risk factors for offending against children, and potential points for intervention, disruption and prevention of abuse. Data were drawn from three quota-based samples of men aged 18 years or over comparable to the Australian, U.K., and U.S. male populations in terms of age, residential region, annual household income, and educational attainment. Survey recruitment and administration were conducted by CloudResearch (https://www.cloudresearch.com), an online research panel company with access to an international pool of over 1.5 million participants. Eligible participants within this pool were notified based on their demographic profiles when the survey project was launched and could elect whether or not to participate. Participants were informed that the aim of the study was to “measure the prevalence of online child sexual exploitation offending, risk behaviours and attitudes amongst Australian, U.S., and U.K. males aged 18 and above” and that they had been selected to participate because they lived in the countries of interest and were over the age of consent. The survey was reviewed by a project advisory group that included representatives from law enforcement, financial intelligence units, government departments, and mental health support services. Whereas most surveys on sexual interest and behaviour towards children amongst people in the community have focused on psychological factors and instruments, with an eye to informing treatment, this survey was developed to examine demographic, behavioural and attitudinal correlates of child sex offending to inform prevention, disruption and public policy responses. Ethical approval for this study was provided by the Human Research Ethics Committee at the University of New South Wales (HC220317).

A total of 7,343 individuals consented to participate in the study (Australia *n* = 2,703; United Kingdom *n* = 2,243; United States *n* = 2,397), of which 6,577 completed the survey. From this, 1,591 participants were removed because they indicated being female at birth, did not identify as male, failed the mid-survey attention check, or reported not answering honestly. An additional 68 participants were excluded due to missing data on demographic benchmark variables required for data weighting. The final analytical sample consisted of 4,918 participants (Australia: 1,939; United Kingdom: 1,506; United States: 1,473). Next, iterative proportional fitting was conducted to improve sample representativeness in terms of age, annual household income, race/cultural background, educational attainment, marital status, and workforce participation. Benchmark weights were based on census data of Australian males aged 15 years and over (Australian Bureau of Statistics, 2021), U.K. males aged 16 years and over (Office for National Statistics, 2021), and U.S. males aged 18 years or over (U.S Census Bureau, 2021). Participants were required to answer all non-demographic questions before proceeding to the next set of questions. However, for certain questions related to child sexual abuse, an “unsure” response option was available. For the current study, data from the three countries were pooled (*n* = 4,918) to increase statistical power.

### Measures

#### Sexual Interest in Children

Participants were coded as having sexual interest in children if they answered yes to any of the following questions: (a) I would have sexual contact with a child between 12 and 14 years if no one would find out; (b) I would have sexual contact with a child between 10 and 12 years if no one would find out; (c) I would have sexual contact with a child younger than 10 years if no one would find out; (d) the lowest age I typically find attractive is 15 years or younger; (e) the highest age I typically find attractive is 15 years or younger; and (f) I have concerns about my sexual feelings towards people below the age of 18 and would like more information and support. Respondents who indicated yes to any of these questions were coded as having sexual interest in children. These questions were developed from other measures of sexual interest deployed in Briere and Runtz (1989) and Ó Ciardha et al. (2022).

#### Child Sex Offending

Participants were asked if they had engaged in any of the following online behaviours while over the age of 18 years: (a) knowingly and deliberately viewed pornographic material containing people below the age of 18 (referred to as CSAM); (b) flirted or had sexual conversations with a person below the age of 18 online; (c) engaged in a sexually explicit webcam interaction with a person below the age of 18; (d) paid for online sexual interactions, images, or videos involving a person below the age of 18, and (e) while over the age of 18 years, ever having had sex or sexual contact with a person below the age of 18 years. These measures were developed using a variety of validated survey measures, and new questions were created where measures were not available (Austin & Salter, 2024). Respondents who indicated yes to any of these questions were coded as having engaged in child sex offending.

#### Demographic Characteristics

Participants reported their sexual orientation (0 = *not heterosexual*; 1 = *heterosexual*), if they had ever had sex with men (0 = *no*; 1 = *yes*), current relationship status (0 = *single, widowed, divorced, or separated*; 1 = *married or living with partner*), employment over the last 3 months (0 = *unemployed*; 1 = *casual, part-time, or full-time employment*), highest educational attainment (0 = *did not obtain a bachelor’s degree*; 1 = *bachelor’s degree or higher*), number of children living in the household (0 = *none*; 1 = *one or more*), if their current work involves contact with children (0 = *does not work with children*; 1 = *works with children*), age (1 = *18–34 years*; 2 = *35–64 years*; 3 = *65 years or older*), total US standardised household income before taxes during the last 12 months (1 = *less than US$25,000 equivalent*; 2 = *between US$25,000–US$99,999*; 3 = *US$100,000 or more*), and residential location (1 = *city*; 2 = *suburb*; 3 = *rural or regional*).

#### Online Pornography Habits

Respondents indicated (0 = *no*; 1 = *yes*) if, while over the age of 18 years, they: (a) knowingly and deliberately viewed pornography involving sex between humans and animals; (b) watched pornography that included sex with violence or force; and (c) purchased online sexual services from another person. Questions about adult pornography were included in the survey since extensive consumption of adult pornography has been identified as a common antecedent or correlate of technology-facilitated child sex offending (Wortley et al., 2024). The survey included specific questions about sadism and bestiality, since these are characteristics of highly traded CSAM (Seto et al., 2018). Feedback from members of the project advisory group indicated that payment for online sexual services was, anecdotally, a feature of investigations into technology-facilitated child sexual abuse, and so a question on this topic was added to explore the hypothesis that payment for online sexual services is higher amongst men who commit offences against children.

#### Frequency of Online Activities

Participants reported how often (1 = *never*, 2 = *less than monthly*, 3 = *monthly*, 4 = *weekly*, 5 = *daily*) they engaged in 13 common internet activities: (a) using search engines to browse or look for information; (b) sending emails; (c) using social media; (d) engage in online blogs; (e) shopping online; (f) online banking; (g) online messaging; (h) private video chatting; (i) livestreaming self; (j) streaming movies or shows to personal devices; (k) using romance websites or dating apps; (l) online gaming; and (m) watching online pornography. These questions were asked since there is relatively limited data on the routine (rather than offence-related) online activities of child sex offenders that might provide evidence on similarities or differences in their use of online platforms and services compared to other populations. Such information may be useful for early intervention or secondary prevention services seeking to more effectively target or intersect with men with a sexual interest in children, in order to encourage them to access help and support options.

#### Social Media Platform Use

Participants indicated (0 = *no*, 1 = *yes*) if they currently used any of the following social media platforms: (a) YouTube; (b) Instagram; (c) Facebook; (d) Snapchat; (e) Facebook Messenger; (f) TikTok; (g) WhatsApp; (h) Twitter; (i) Discord; (j) Skype; and (k) Viber. Given the outsized role of social media platforms in the online grooming, solicitation, and exploitation of children, as well as networking between offenders (Thiel et al., 2023), these questions were designed to provide data on the extent of offender engagement across a range of social media services.

#### Mental Health and Social Supports

Symptoms of anxiety and depression were measured using four items from the Patient Health Questionnaire 4 (PHQ-4; Löwe et al., 2010). The PHQ-4 asks respondents how often (1 = *not at all*; 2 = *1–7 days*; 3 = *8–11* *days*; 4 = *12–14 days*) over the past 2 weeks had they been bothered by: (a) little interest or pleasure in doing things; (b) feeling down, depressed, or hopeless; (c) feeling nervous, anxious, or on edge; and (d) not being able to stop or control worrying. Scores were summed so that higher scores reflect greater frequency of anxiety and depression symptoms (α = .89). Substance misuse frequency was assessed using four questions from the National Institute on Drug Abuse Quick Screen v1.0 (http://www.drugabuse.gov/nmassist/). Participants indicated how often (1 = *never*; 5 = *daily or almost daily*) over the past year they: (a) drank five or more alcoholic drinks a day (i.e. binge drink); (b) used tobacco products; (c) used prescription drugs for non-medical reasons; and (d) used illicit drugs. The availability of social supports was measured using the Multidimensional Scale of Perceived Social Support (MSPSS; Zimet et al., 1990). The MSPSS includes 12 questions responded to on a 7-point Likert scale (1 = *very strongly disagree*; 7 = *very strongly agree*) that correspond to social support from significant others (α = .94), family (α = .92), and friends (α = .93). These questions are relevant since existing research indicates that offending risk is influenced by negative mental states, such as depression, as well as the effects of alcohol and drugs (Wortley et al., 2024). Reducing these risk factors and improving social supports are key goals of early intervention and prevention services (Wilpert & Janssen, 2020).

#### Adverse Childhood Experiences

Participants reported if, any time prior to the age of 18 years, they experienced emotional abuse, physical abuse, sexual abuse, low family support, neglect, parental divorce, domestic violence, household drug abuse, household mental illness, or household member incarcerated (Felitti et al., 1998). Adverse childhood experiences are elevated in offending populations, including amongst sex offenders (Levenson & Socia, 2016).

#### Attitudes Towards Child Sexual Abuse

Twenty-five items adapted from the Child Sexual Abuse Myth Scale were used to measure men’s attitudes towards online child sexual exploitation (see Supplemental Materials). The scores for each item ranged from *strongly disagree* (1) to *strongly agree* (5). Principal components analysis was conducted to reduce the 25 items into multiple subscales reflecting different underlying dimensions (Salter et al., forthcoming). Four components were identified, one of which only comprised of two items (α = .73) and was not retained. The three remaining components were designated “denial of abusiveness” (14 items, α = .92), “normalisation/blame diffusion” (5 items, α = .87), and “restrictive stereotypes” (4 items, α = .81). The average score for each subscale was calculated, with higher scores indicating greater endorsement of attitudes. “Denial of abusiveness” refers to attitudes or beliefs that child sexual abuse is a benign or positive experience, and that children can consent to being abused by adults. “Normalisation/blame diffusion” refers to the belief that technology-facilitated child sexual exploitation is acceptable, including in the presence of potentially mitigating factors. “Restrictive stereotypes” involves beliefs that deny the facts of abuse and minimise the undesirable consequences of abuse. Although these items may not represent stereotypes in a conventional demographic or social sense, they align conceptually with the original Collings (1997) scale, in which distorted or narrowing explanations for CSA (e.g. linking abuse to poverty or stigma) were interpreted as indicative of stereotyped or minimising beliefs.

### Analytical Strategy

The analyses for this exploratory study were based on the pooled subgroup of 642 men from Australia (*n* = 236, 12.2%), United Kingdom (*n* = 157, 10.4%), and the United States (*n* = 249, 16.9%) who had sexual interest in children. First presented are the descriptive statistics regarding online child sex offending, demographic characteristics, frequency of online activities, use of social media platforms, mental health and social supports, adverse childhood experiences, and attitudes towards child sexual abuse, among men with sexual interest in children for the pooled sample and separately for the Australian, U.K., and U.S. samples. Next, logistic regression analyses were conducted based on the pooled sample, and adjusted for country, to examine the variables associated with wanting help for sexual interest in children, relative to men who did not want help for their sexual interest in children. Associations were considered significant if *p*-values were *p* < .01. Results were calculated using survey weights, with standard errors adjusted to consider the complex sample design (Lumley, 2004). Odds ratios (OR) and 99% confidence intervals (99% CIs) were reported as measures of effect size and precision of the association between the covariates and outcome variables. For categorical measures, the OR indicates how the odds of an outcome differ between a specific category and a reference category, while for continuous measures, the OR reflects the change in odds associated with a one-unit increase in the predictor variable. Analyses were conducted using IBM SPSS version 29 (IBM Corp, 2022).

## Results

Of the 4,918 men in the pooled sample, around one-in-seven (642, 13.1%) had sexual interest in children. Men who had sexual interest in children comprised 12.2% of the Australian sample, 10.4% of the U.K. sample, and 16.9% of the U.S. sample (*x*^2^ (2, *n* = 4,918) = 30.23, *p* < .001). The descriptives presented in Table 1 indicate that just under half (43.8%) of all men who had sexual interest in children wanted help for their interest. This proportion was significantly greater for those from the United States (52.0%) than Australia (37.0%) (*x*^2^ (1, *n* = 485) = 11.98, *p* = .005), but not from the United Kingdom (41.1%) (*x*^2^ (1, *n* = 406) = 4.31, *p* = .066). Furthermore, almost half (41.9%) of those who had sexual interest in children had engaged in online child sex offending, with this proportion being significantly lower for the Australian (30.6%) than the U.S. (49.9%) (*x*^2^ (1, *n* = 485) = 20.24, *p* < .001) and U.K. (46.3%) sample (*x*^2^ (1, *n* = 393) = 11.11, *p* = .007).

**Table 1.** Weighted Descriptive Statistics (%/Mean [99% CI]) for Men Who Have Sexual Feelings Towards Children (*n* = 642).

Measures	Pooled (*n* = 642), % /m	Australia (*n* = 236), % /m	United Kingdom (*n* = 157), % /m	United States (*n* = 249), % /m
Want help for feelings (%)	43.8 [38.0, 49.9]	37.0 [29.9, 44.7]	41.1 [32.5, 50.4]	52.0 [45.0, 59.0]
Child sex offending (%)				
Any offending	41.9 [36.2, 47.9]	30.6 [22.4, 40.3]	46.3 [34.6, 58.4]	49.9 [40.7, 59.0]
Any offline offending	17.3 [13.4, 22.0]	10.2 [6.3, 16.3]	21.0 [13.0, 32.1]	21.6 [14.9, 30.4]
Any online offending	36.2 [30.7, 42.1]	28.0 [21.8, 35.2]	38.4 [29.9, 47.7]	42.6 [35.8, 49.7]
Watch CSAM	19.5 [15.2, 24.6]	15.6 [10.9, 21.8]	19.2 [12.8, 27.7]	23.4 [17.9, 29.9]
Sexual conversations with children online	16.6 [12.8, 21.4]	11.0 [7.2, 16.3]	16.5 [10.8, 24.4]	22.1 [16.9, 28.3]
Sexually explicit webcam with child online	12.8 [9.5, 17.1]	7.2 [4.5, 11.3]	9.8 [5.6, 16.6]	20.1 [15.0, 26.3]
Paid for sexual content of children online	15.5 [11.8, 20.3]	8.8 [5.7, 13.5]	14.8 [9.3, 22.8]	22.3 [17.0, 28.8]
Online pornography habits (%)				
Watches violent and/or rough sexual content	22.2 [17.7, 27.5]	20.7 [15.2, 27.5]	19.0 [12.7, 27.3]	25.6 [20.0, 32.1]
Watches bestiality	17.3 [13.2, 22.3]	14.4 [9.6, 21.0]	12.2 [7.4, 19.7]	23.3 [17.9, 29.7]
Purchase adult sexual content	33.2 [28.0, 39.0]	20.6 [15.4, 26.9]	33.3 [25.3, 42.4]	45.2 [38.3, 52.3]
Mean attitudes towards child sex abuse				
Denial of abusiveness	2.79 [2.67, 2.91]	2.50 [2.36, 2.64]	2.76 [2.59, 2.93]	3.07 [2.92, 3.22]
Normalisation/blame diffusion	2.65 [2.53, 2.77]	2.96 [2.79, 3.14]	2.52 [2.36, 2.68]	2.43 [2.30, 2.56]
Restrictive stereotypes	2.97 [2.85, 3.10]	2.72 [2.57, 2.87]	2.94 [2.78, 3.11]	3.24 [3.09, 3.38]
Demographic characteristics (%)				
Heterosexual	93.3 [89.5, 95.8]	94.9 [89.5, 97.6]	92.2 [85.9, 95.9]	92.5 [87.9, 95.5]
Had sex with men	33.6 [28.1, 39.4]	29.3 [22.8, 36.8]	38.8 [30.1, 48.3]	34.3 [28.0, 41.2]
Married or living with partner	63.6 [57.4, 69.4]	64.6 [56.2, 72.1]	55.3 [46.0, 64.3]	67.9 [60.8, 74.2]
Bachelor’s degree or higher	44.0 [38.2, 50.0]	40.8 [33.5, 48.6]	48.4 [39.3, 57.6]	44.2 [37.4, 51.2]
Employed	74.7 [68.8, 79.9]	71.4 [63.1, 78.5]	78.7 [69.8, 85.5]	75.4 [68.5, 81.1]
Child in household	49.7 [43.7, 55.6]	40.8 [33.5, 48.5]	50.0 [40.9, 59.2]	57.8 [50.7, 64.6]
Works with children	28.6 [23.7, 34.0]	18.4 [14.1, 23.6]	35.1 [26.8, 44.3]	34.2 [27.9, 41.1]
Residential location				
City	60.6 [54.5, 66.4]	55.0 [47.0, 62.7]	58.9 [49.6, 67.7]	67.0 [60.1, 73.2]
Suburbs	28.1 [22.8, 34.0]	34.5 [27.2, 42.6]	26.2 [18.7, 35.4]	23.1 [17.7, 29.6]
Regional or rural	11.3 [8.1, 15.7]	10.6 [6.5, 16.8]	14.8 [9.5, 22.5]	9.9 [6.6, 14.6]
Age				
18–34 years	47.0 [41.1, 53.1]	45.8 [38.1, 53.6]	52.5 [43.3, 61.5]	44.7 [37.8, 51.9]
35–64 years	40.5 [34.7, 46.5]	31.3 [24.3, 39.4]	38.5 [30.0, 47.7]	50.3 [43.3, 57.3]
65 years and older	12.5 [9.3, 16.7]	22.9 [17.3, 29.6]	9.0 [5.2, 15.2]	4.9 [2.8, 8.6]
Household income				
Less than US$25,000	23.4 [18.4, 29.4]	26.1 [19.1, 34.7]	20.2 [13.8, 28.5]	22.9 [17.3, 29.8]
US$25,000–US$99,999	43.6 [37.8, 49.6]	36.1 [29.2, 43.6]	65.3 [56.7, 73.1]	37.0 [30.7, 43.8]
US$100,000 or more	33.0 [27.7, 38.7]	37.8 [30.7, 45.4]	14.5 [10.4, 19.8]	40.1 [33.4, 47.2]
Mean frequency of online activities				
Browse online	4.44 [4.32, 4.56]	4.30 [4.10, 4.50]	4.52 [4.39, 4.65]	4.52 [4.40, 4.64]
Send emails	4.04 [3.88, 4.20]	3.93 [3.70, 4.17]	4.12 [3.93, 4.32]	4.09 [3.91, 4.27]
Social media	4.27 [4.13, 4.41]	4.20 [4.00, 4.40]	4.16 [3.95, 4.37]	4.41 [4.26, 4.57]
Online blogs	3.55 [3.36, 3.73]	3.28 [3.03, 3.53]	3.50 [3.24, 3.77]	3.83 [3.62, 4.04]
Online shopping	3.52 [3.37, 3.67]	3.29 [3.09, 3.48]	3.45 [3.25, 3.65]	3.79 [3.61, 3.96]
Online banking	3.92 [3.79, 4.05]	3.86 [3.70, 4.02]	3.99 [3.82, 4.17]	3.94 [3.77, 4.11]
Online messaging	3.98 [3.83, 4.14]	3.82 [3.61, 4.04]	3.98 [3.77, 4.19]	4.13 [3.96, 4.31]
Private video chatting	3.47 [3.30, 3.64]	3.20 [2.98, 3.43]	3.61 [3.39, 3.83]	3.63 [3.43, 3.83]
Livestream self	3.43 [3.24, 3.62]	3.17 [2.92, 3.41]	3.30 [3.02, 3.58]	3.75 [3.53, 3.97]
Streaming videos	3.89 [3.73, 4.06]	3.56 [3.33, 3.80]	3.95 [3.75, 4.15]	4.17 [4.00, 4.34]
Romance/dating websites	2.99 [2.80, 3.19]	2.63 [2.40, 2.87]	2.99 [2.70, 3.28]	3.33 [3.10, 3.57]
Online gaming	3.18 [3.00, 3.36]	2.99 [2.75, 3.22]	2.98 [2.72, 3.24]	3.48 [3.28, 3.68]
Online pornography	3.15 [2.96, 3.34]	2.94 [2.70, 3.18]	3.12 [2.84, 3.40]	3.36 [3.14, 3.58]
Use of social media platforms (%)				
YouTube	80.4 [75.2, 84.7]	77.4 [69.9, 83.5]	79.3 [71.2, 85.6]	83.8 [78.3, 88.1]
Instagram	59.1 [53.0, 64.9]	51.0 [43.1, 58.7]	57.3 [48.1, 66.1]	67.8 [60.9, 74.0]
Facebook	70.8 [65.1, 75.9]	68.4 [60.9, 75.0]	67.7 [58.6, 75.6]	75.0 [68.2, 80.7]
Snapchat	42.8 [37.0, 48.8]	35.3 [28.4, 42.8]	43.2 [34.2, 52.6]	49.7 [42.7, 56.7]
Facebook Messenger	48.0 [42.1, 54.0]	40.4 [33.0, 48.3]	49.8 [40.7, 59.0]	54.1 [47.0, 61.0]
TikTok	44.0 [38.1, 50.0]	32.7 [25.7, 40.6]	44.9 [35.8, 54.3]	54.0 [47.0, 60.9]
WhatsApp	49.8 [43.8, 55.8]	39.6 [32.3, 47.4]	65.8 [57.0, 73.6]	49.4 [42.4, 56.4]
Twitter	43.3 [37.4, 49.3]	34.8 [27.9, 42.4]	40.3 [31.5, 49.7]	53.1 [46.1, 60.1]
Discord	17.8 [13.7, 22.7]	15.5 [10.8, 21.7]	18.6 [12.6, 26.6]	19.4 [14.5, 25.6]
Skype	22.8 [18.3, 28.0]	18.7 [14.0, 24.5]	25.0 [17.8, 33.9]	25.2 [19.7, 31.8]
Viber	8.0 [5.2, 12.2]	6.6 [3.5, 12.4]	9.5 [5.1, 17.1]	8.4 [5.2, 13.3]
Mean mental health and social support				
Binge drinking frequency	2.69 [2.53, 2.85]	2.43 [2.25, 2.62]	2.77 [2.53, 3.01]	2.89 [2.69, 3.09]
Smoking frequency	2.72 [2.53, 2.90]	2.26 [2.05, 2.48]	2.82 [2.54, 3.09]	3.08 [2.86, 3.30]
Prescription drug misuse frequency	2.31 [2.15, 2.47]	1.98 [1.82, 2.14]	2.28 [2.03, 2.53]	2.64 [2.44, 2.84]
Illegal drug use frequency	2.06 [1.90, 2.22]	1.76 [91.60, 1.92]	2.09 [1.83, 2.35]	2.33 [2.12, 2.53]
Anxiety & Depression (PHQ-4)	3.94 [3.57, 4.32]	3.47 [3.05, 3.88]	3.88 [3.26, 4.50]	4.44 [3.97, 4.91]
Support from significant others	4.83 [4.62, 5.04]	4.57 [4.28, 4.86]	4.62 [4.33, 4.91]	5.20 [4.98, 5.42]
Support from family	4.80 [4.59, 5.00]	4.60 [4.31, 4.89]	4.63 [4.36, 4.91]	5.08 [4.86, 5.30]
Support from friends	4.80 [4.60, 5.00]	4.58 [4.31, 4.86]	4.60 [4.34, 4.87]	5.13 [4.91, 5.35]
Adverse Childhood Experiences (%)				
Childhood emotional abuse	38.2 [32.6, 44.1]	27.5 [21.2, 34.9]	41.9 [33.1, 51.2]	46.0 [39.1, 53.1]
Childhood physical abuse	34.4 [29.0, 40.3]	24.0 [18.0, 31.2]	37.0 [28.6, 46.4]	42.7 [35.9, 49.8]
Childhood sexual abuse	29.4 [24.3, 35.1]	19.2 [13.9, 25.8]	35.2 [26.9, 44.5]	35.4 [29.1, 42.3]
Childhood low family support	31.8 [26.6, 37.6]	21.8 [16.4, 28.5]	36.9 [28.5, 46.2]	38.1 [31.5, 45.1]
Childhood neglect	26.0 [21.2, 31.5]	16.8 [12.0, 22.9]	24.7 [17.6, 33.5]	35.5 [29.1, 42.5]
Childhood parental divorce	31.5 [26.2, 37.5]	24.5 [18.3, 32.0]	30.4 [22.8, 39.4]	38.8 [32.2, 46.0]
Childhood domestic violence	21.7 [17.1, 27.0]	14.6 [9.9, 21.0]	22.1 [15.3, 30.9]	28.5 [22.1, 34.9]
Childhood family drug use	28.5 [23.4, 34.2]	24.0 [18.0, 31.1]	20.3 [13.7, 28.9]	38.0 [31.3, 45.1]
Childhood family mental illness	22.8 [18.3, 28.1]	19.2 [13.9, 25.8]	19.1 [13.1, 27.0]	28.6 [22.7, 35.3]
Childhood family incarceration	21.6 [17.0, 26.9]	13.4 [8.6, 20.2]	17.4 [11.8, 24.9]	32.0 [25.7, 38.9]

*Note.* CSAM = child sexual abuse material; PHQ-4 = Patient Health Questionnaire 4.

Table 2 presents the country-adjusted odds of wanting help for sexual interest in children by engagement in child sex offending, online pornography habits, and attitudes towards child sex abuse, relative to men who did not want help for their sexual interest in children. Men who reported wanting help for their sexual feelings had 2.78 times the odds of having engaged in *any* form of child sex offending, compared to those who did not want help. This association appears to be driven by significantly higher odds of engagement in online child sex offending but not sexual contact with a child. Specifically, men who wanted help for their sexual interest in children were twice as likely to watch online CSAM, and around three times as likely to have a sexual conversation or webcam with a child or pay for sexual content of a child online. The odds of men wanting help for their sexual interest in children were also around two to two-and-a-half times higher if they watched violent and/or rough sexual content (OR = 2.26), bestiality (OR = 2.74), and purchased sexually explicit adult content online (OR = 1.86). In terms of attitudes towards child sex abuse, men who wanted help for their sexual interest in children had lower odds of endorsing “blame diffusion” attitudes, and significantly higher odds of endorsing “restrictive stereotypes,” compared to men who did not want help for their sexual feelings.

**Table 2.** Country Adjusted Weighted Odds (99% CI) of Wanting Help for Sexual Feelings Towards Children by Engagement in Child Sex Offending, Porn Usage, and Attitudes Towards Child Sex Abuse (*n* = 642).

Measures	OR	99% CI
Child sex offending		
Any offending	2.78	[1.68, 4.59]
Any offline offending	1.68	[0.91, 3.09]
Any online offending	2.72	[1.64, 4.52]
Watch CSAM	2.10	[1.14, 3.89]
Sexual conversations	2.92	[1.56, 5.47]
Sexually explicit webcam	3.40	[1.64, 7.05]
Paid for sexual content	2.92	[1.47, 5.78]
Online pornography habits		
Watches violent and/or rough sexual content	2.26	[1.27, 4.03]
Watches bestiality	2.74	[1.40, 5.37]
Purchase adult sexual content	1.86	[1.12, 3.10]
Attitudes towards child sex abuse		
Denial of abusiveness	1.12	[0.87, 1.45]
Normalisation/blame diffusion	0.56	[0.44, 0.73]
Restrictive stereotypes	1.31	[1.04, 1.66]

*Note.* CSAM = child sexual abuse material.

As reported in Table 3, there were few demographic factors differentiating men who did and did not want help for their sexual interest in children. Men who wanted help for their sexual interest in children had 1.75 times the odds of being married or living with their partner, and 1.83 times the odds of working with children, compared to men who did not want help.

**Table 3.** Country Adjusted Weighted Odds (99% CI) of Wanting Help for Sexual Feelings Towards Children by Demographic Characteristics (*n* = 642).

Measures	OR	99% CI
Heterosexual	1.40	[0.55, 3.62]
Had sex with men	0.89	[0.53, 1.47]
Married or living with partner	1.75	[1.01, 3.04]
Bachelor’s degree or higher	1.56	[0.96, 2.54]
Employed	1.13	[0.62, 2.07]
Child in household	1.16	[0.71, 1.88]
Works with children	1.83	[1.09, 3.06]
Residential location		
City	1.17	[0.66, 2.09]
Suburbs	1.00 (reference)	
Regional or rural	1.47	[0.59, 3.65]
Age		
18–34 years	0.79	[0.46, 1.35]
35–64 years	1.00 (reference)	
65 years and older	1.49	[0.68, 3.26]
Household income		
Less than US$25,000	1.00 (reference)	
US$25,000–US$99,999	1.47	[0.74, 2.93]
US$100,000 or more	1.63	[0.81, 3.30]

Table 4 indicates that the frequency of engagement in various online activities – measured on a 5-point scale (1 = *never*, 5 = *daily*) – was generally not significantly associated with wanting help for sexual interest in children. The exception to this was online banking and online messaging, where each one-unit increase in frequency was associated with 1.25 and 1.27 times greater odds of wanting help, respectively. For example, assuming a linear association, a man who uses online messaging daily has 6.35 times the odds of wanting help for his sexual interest in children compared to someone who never uses online messaging. The same table also demonstrates that men who wanted help for their sexual interest in children had significantly greater odds of using certain social media platforms (measured on a dichotomous scale of 0 = *no*, 1 = *yes*) than men who did not want help for their sexual interest in children. These were specifically Viber (OR = 2.78),^2^ Skype (OR = 2.08), WhatsApp (OR = 2.05), YouTube (OR = 1.92), and Twitter (OR = 1.63).

**Table 4.** Country Adjusted Weighted Odds (99% CI) of Wanting Help for Sexual Feelings Towards Children by Frequency of Online Activities and Use of Social Media Platforms (*n* = 642).

Measures	OR	99% CI
Online activities frequency		
Browse online	1.23	[0.91, 1.66]
Send emails	1.23	[0.97, 1.57]
Social media	1.13	[0.91, 1.41]
Online blogs	1.18	[0.98, 1.41]
Online shopping	1.09	[0.88, 1.36]
Online banking	1.25	[1.00, 1.57]
Online messaging	1.27	[1.03, 1.57]
Private video chatting	1.08	[0.89, 1.31]
Livestream self	1.12	[0.94, 1.33]
Streaming videos	1.17	[0.94, 1.44]
Romance/dating websites	1.01	[0.85, 1.19]
Online gaming	1.13	[0.95, 1.36]
Online pornography	1.12	[0.94, 1.34]
Use of social media platforms		
YouTube	1.92	[1.04, 3.55]
Instagram	1.23	[0.74, 2.04]
Facebook	1.30	[0.76, 2.24]
Snapchat	1.34	[0.82, 2.18]
Facebook Messenger	1.36	[0.83, 2.22]
TikTok	1.23	[0.75, 2.03]
WhatsApp	2.05	[1.24, 3.38]
Twitter	1.63	[1.00, 2.68]
Discord	1.28	[0.71, 2.32]
Skype	2.08	[1.19, 3.64]
Viber	2.78	[1.00, 7.72]

Table 5 presents the odds for mental health and social supports; all measured on continuous scales. The odds of men wanting help for their sexual interest in children were 1.22 times greater for each one-unit increase in binge drinking frequency. The odds of wanting help were not significantly associated with misuse of other substances, symptoms of depression and anxiety, or a history of adverse childhood experiences, compared to men who did not want help for their sexual interest in children. However, those who wanted help for their feelings had significantly greater odds of perceived social support from significant others (OR = 1.32), family (OR = 1.33), and friends (OR = 1.30).

**Table 5.** Country Adjusted Weighted Odds (99% CI) of Wanting Help for Sexual Feelings Towards Children by Mental Health, Social Supports, and Adverse Childhood Experiences (*n* = 642).

Measures	OR	99% CI
Mental health		
Binge drinking frequency	1.22	[1.00, 1.48]
Smoking frequency	1.07	[0.90, 1.27]
Prescription drug misuse frequency	1.07	[0.89, 1.28]
Illegal drug use frequency	1.06	[0.90, 1.26]
Anxiety & Depression (PHQ-4)	1.01	[0.94, 1.09]
Social support		
Support from significant others	1.32	[1.12, 1.57]
Support from family	1.33	[1.12, 1.58]
Support from friends	1.30	[1.09, 1.54]
Adverse childhood experiences		
Childhood emotional abuse	1.28	[0.78, 2.10]
Childhood physical abuse	1.06	[0.64, 1.76]
Childhood sexual abuse	1.48	[0.87, 2.51]
Childhood low family support	1.26	[0.76, 2.11]
Childhood neglect	1.20	[0.70, 2.08]
Childhood parental divorce	1.10	[0.64, 1.86]
Childhood domestic violence	1.42	[0.80, 2.57]
Childhood family drug use	1.25	[0.73, 2.16]
Childhood family mental illness	1.38	[0.79, 2.43]
Childhood family incarceration	1.41	[0.77, 2.56]

*Note.* PHQ-4 = Patient Health Questionnaire 4.

## Discussion

This paper found that one in seven men in the survey reported some sexual interest in children, a finding broadly in line with other prevalence studies on sexual interest in children (Savoie et al., 2021). Importantly, this proportion varied considerably between countries and was highest in the United States. The United States also had the highest proportion of men with sexual interest in children who had offended, and who wanted help, compared to the two other jurisdictions. This finding points to the fact that rates of sexual interest in children are not consistent across jurisdictions and appear to vary according to social, cultural and policy context, a point taken up in other articles in this special issue. This finding is important for the purposes of child abuse prevention, since it suggests that the proportion of people in the community with a sexual interest in children may increase or decrease depending on developmental and environmental influences and/or social structural factors. Amidst expanding interest in the determinants of child sexual abuse victimisation (Turner et al., 2023), these findings suggest that the social, economic and commercial determinants of the *perpetration* of sexual violence against children also deserve further study.

Compared to the other two jurisdictions, proportionately fewer men with a sexual interest in children in Australia had engaged in offending. This finding is interesting because, of the three jurisdictions, only the United Kingdom has had nationally accessible prevention or early intervention services in place for any length of time (De Boeck et al., 2022). In comparison, such services have been largely unavailable in Australia until recently (this service remains in limited pilot form at the time of writing), while prevention/early intervention has been infrequently offered in limited locations throughout the United States. We might therefore expect to see lower rates of offending amongst men with a sexual interest in children in the United Kingdom since this population has been offered early intervention services for over 2 decades. The finding of this study that offending amongst men with a sexual interest in children is lower in Australia than the United Kingdom may have a number of explanations, including that the variance and impact of the social or contextual determinants of sexual interest in children and/or offending may exceed the community-level impact of early intervention services. This finding may also point to the challenges of engaging early in the offending trajectories of people with a sexual interest in children, and the importance of scaling up prevention and early intervention strategies.

Just over 40% of men with sexual interest in children wanted help for those feelings. There were few demographic differences between men who did and did not want help for their sexual interest in children. The key demographic difference was that men with sexual interest in children who did want help were almost twice as likely to be married or living with a partner or working which children. They also reported significantly higher perceived social support from significant others, family and friends. While men with sexual interest in children who wanted help were somewhat more likely to binge drink, mental health status and history of childhood adversity were similar between the two groups. The major distinguishing factor between these two groups was that, compared to men with towards children who did not want help, men with sexual interest in children who did want help were almost three times more likely to have engaged in any form of child sex offending. Their offending was predominantly online (such as viewing CSAM, grooming children, or paying for sexual content of children). They also evinced a pattern of sexual arousal to other deviant material: they were over twice as likely to watch violent adult pornography, and almost three times more likely to view bestiality material. Their elevated consumption of violent and bestiality pornography suggests that their sexual interest in children clusters with other paraphilic interests, and may be indicative of hypersexuality, which has been consistently identified as a risk factor for child sex offending (Wurtele et al., 2018).

Media and academic depictions of people struggling with sexual interest in children, who are motivated to seek help, have often focused on the intrinsic moral conflict posed by their sexual impulses (Malone, 2016). However, our paper does not suggest that men who wanted help for their sexual interest in children were motivated primarily by moral or ethical concern over child sexual abuse. However, this group were more likely to endorse stereotypical excuses for child sexual abuse than men with sexual feelings who do not want help. Such excuses and rationalisations have been documented elsewhere in research on cognitive distortions amongst child sex offenders (Kåven et al., 2019). Given that men with sexual interest in children who wanted help were more likely to be offending, and were also more likely to be married or partnered, with good social networks, they may be motivated to seek help out of concern about the impact on their relationships and reputation if their offending behaviour is uncovered. There is relatively little research into men’s motivations for voluntarily seeking help for their perpetration of violence and abuse, since most perpetrator interventions are situated within the criminal justice system and have a high degree of mandated attendance. Research has consistently found that, at least for some men, program attendance and willingness to change is linked to the hope that they might protect their families and relationships from their abusive behaviour (McGinn et al., 2016; Stanley et al., 2010; Turhan & Bernard, 2022).

While prior research has focused on domestic violence offender treatment, the findings of this paper suggest that similar motivations may be at work amongst men with a sexual interest in children who want help. Nonetheless, this group was also more likely to be working with children compared to men with sexual feelings who did not want help. They were also more likely to use encrypted services such as Viber, Skype and WhatsApp compared to men with a sexual interest in children who do not want help. These findings may indicate a degree of premeditation in their offending against children, including seeking child-focused employment and preferring online services that camouflage illegal activities. This finding is indicative of their risk to children and the urgent need for intervention, but it may also signal their awareness of the risk they pose to the children they are in contact with.

## Conclusion

This is the first paper to delineate between men with sexual interest in children who do want help, and those who do not. Consistent with the evaluations of secondary prevention programs (Horn et al., 2015), these findings suggest that offending behaviour is a key motivator for help-seeking amongst men with sexual interest in children. These men also displayed heightened rates of other forms of sexual deviancy, such as arousal to bestiality pornography, as well as the use of encrypted social media apps, potentially to camouflage or hide their offending activity. Given that help-seeking men were more likely to be married or living with a partner and reported closer social bonds compared to other men, they may be particularly concerned about the impact of their offending (and other deviant online behaviours and interests) on their close relationships, social ties and reputation. Men with sexual feelings who wanted help were also more likely to be working with children than men with sexual feelings who do not want help, which reinforces the importance of prevention and risk minimisation service delivery to this group, as well as law enforcement action to detect any current offending.

These findings have a number of implications for secondary prevention efforts. Firstly, the variation between countries in the proportion of men with a sexual interest in children indicates that sexual interest in children is not uniformly distributed amongst men, but varies significantly between jurisdictions on the basis of social and contextual determinants. This is an important finding for prevention advocates and services, since it indicates that sexual interest in children is not a fixed problem but is responsive to external influences. Second, increasing motivation amongst men who do not want help is an important goal for secondary prevention services. Our findings suggest that prevention messages with a focus on the impact of child abuse offending on future aspirations, including relationships and professional status, may increase the willingness of men with sexual interest in children to reach out for help at an earlier stage. Third, blame diffusion was more common amongst men with sexual interest in children who did not want help, compared to those who did, indicating that men who did not want help were attributing blame for child sexual abuse to other people and exculpatory factors. Prevention messaging could target these patterns of rationalisation in order to engage more effectively with men with sexual interest in children who do not want help. It is notable that men with sexual interest in children who wanted help were more likely than men with sexual feelings who did not want help to endorse attitudes associated with restrictive stereotypes. This differential may be explained by the fact that the group who wanted help were also more likely to be offending against children, and hence their attitudes reflected and rationalised their pattern of abusive behaviour.

Fourth, while there were not marked differences in social media use between men with sexual interest in children who did or did not want help, men who did want help were more likely to use popular social media sites such as YouTube and Twitter. These findings could help secondary prevention services to more effectively target their outreach to a receptive cohort of men with sexual interest in children. Fifth, the study also found that men with sexual interest in children who wanted help were more likely to evince other problematic behaviours, namely the consumption of violent and deviant pornography. Secondary prevention services, and public health responses to pornography more broadly, could develop messaging to inform pornography consumers about the relationship between consumption of violent and deviant pornography and sexual harm to children, to encourage consumers to engage with secondary prevention services. There is evidence that deterrence or therapeutic-themed messaging targeting pornography consumers seeking to access CSAM is effective at reducing offending or promoting help seeking (Prichard et al., 2024), and such efforts could be expanded to include other problematic pornography genres.

There are a number of limitations to this study. Firstly, the study used 18 as the age of consent for all sexual engagement with children including online offences and sexual contact with children, since this is a comparative international study, and the age of consent and definition of a child varies across criminal offences against children between the three jurisdictions. Accordingly, the study used the definition of a child for the purpose of determining child sexual abuse in accordance with international legal definitions of childhood. The study did not gather specific data on the age at which respondents had sexual contact with a child, since requesting such specific information about potential child sex offences could deter respondents from answering honestly. As a result, it is possible that the study has categorised lawful behaviour as unlawful behaviour in some instances, for example, a 19-year-old having sex with a 17-year-old. In a separate paper (Author, 2025), we provide more information on the participants who reported sexual contact with a child while they were an adult, noting that 71.8% of these men reported other indicators of offending and/or sexual interest in children, including: technology-facilitated sexual offences against a child, age of attraction under 16 years, concern about their sexual feelings towards children, a proclivity to sexually abuse a child under 14 years if they knew they would not be caught, an interest in watching CSAM if they knew they would not be caught, or friends who they knew sexually abused children. The paper reports on the specific characteristics of the remaining third of men who reported sexual contact with a child in adulthood but no other correlates of child sexual interest or offending (Author, 2025). Comparisons with other studies using diverse methodologies indicate that the results of the study are broadly in line with other research on the prevalence of sexual offending and interest in children in community samples (Author, 2025).

A key limitation of this study is that it focused on men with sexual interest in children. Men who sexually offending against children include a significant cohort who do not evince sexual interest in children. Future studies could ask men who report sexual feelings *or* offending against children whether they would like help. This survey focused on men and not women, recognising that the majority of child sex offenders are men, and the survey instrument was developed based on research on male sex offenders. However, research on women’s sexual feelings and offending against children is also important, given the significant minority of female child sex offenders. Such research could shape the outreach of secondary prevention services to potential female clients. This study was conducted in three high-income countries and could be replicated in middle- or low-income countries, where risk factors for sexual feelings and offending against children, and barriers and facilitators to seeking help, are likely to be very different. It is also possible that bias and social desirability may vary between the three jurisdictions, although research on the impact of cultural context on social desirability suggests that “individualistic” countries such as Australia, the United Kingdom and the United States are likely to have lower rates of social desirability bias than “collectivist” cultures” (He et al., 2015). Another limitation of the study is that while the attitudinal components were guided by Collings’ (1997) original Child Sexual Abuse Myth Scale, some adapted items, particularly those in the “restrictive stereotypes” component, may reflect motivational attributions rather than direct minimisation or denial. Further conceptual and psychometric refinement of these components may be warranted in future research.

## Supplemental Material

sj-docx-1-jiv-10.1177_08862605251403618 – Supplemental material for A Descriptive Study of the Attitudes, Characteristics and Behaviours Differentiating Men Who Do and Do Not Want Help for Their Sexual Interest in Children Supplemental material, sj-docx-1-jiv-10.1177_08862605251403618 for A Descriptive Study of the Attitudes, Characteristics and Behaviours Differentiating Men Who Do and Do Not Want Help for Their Sexual Interest in Children by Michael Salter, Delanie Woodlock, Tyson Whitten, Michael Salter, Tyson Whitten, Delanie Woodlock, Mengyao Lu, Maria Lamond, Georgia Naldrett and Matt Tyler in Journal of Interpersonal Violence

## Notes

1. It’s important to note that not all child sex offenders evince a preferential sexual interest in children (Seto, 2019), however, this focus of this paper is on people with a sexual interest in children.

2. The wide confidence intervals associated with Viber use (99% CI [1.00, 7.72]) reflect the relatively small number of users in the analytical sample (*n* = 52, 8.0%), 34 of whom were concerned about their sexual interest in children.
