# Supplementary material for: A Descriptive Study of the Attitudes, Characteristics and Behaviours Differentiating Men Who Do and Do Not Want Help for Their Sexual Interest in Children
Source: J Interpers Violence. 2026 Mar 3;41(7-8):1624–50. doi: 10.1177/08862605251403618 (PMC13009674; doi:10.1177/08862605251403618)
Supplement: sj-docx-1-jiv-10.1177_08862605251403618 – Supplemental material for A Descriptive Study of the Attitudes, Characteristics and Behaviours Differentiating Men Who Do and Do Not Want Help for Their Sexual Interest in Children [file sj-docx-1-jiv-10.1177_08862605251403618.docx]

| Supplemental Table 1. Principal Components Analysis Varimax Rotated Factor Loading from the adapted child sexual abuse myth scale (N = 4,918). | | | | | | |
| --- | --- | --- | --- | --- | --- | --- |
| **#** | **Item** | | **COMPONENT** | | | |
|  |  |  | **1** | **2** | **3** | **4** |
| **COMPONENT 1: Denial of abusiveness** | | |  |  |  |  |
| **1** | Sexual images of a person under 18 online where they seem to be happy and enjoying the activity cannot really be described as ‘abusive’. | | **0.62** | 0.11 | 0.09 | 0.16 |
| **2** | It’s not harmful to look at nude images of someone under 18 if they took the photo of themselves. | | **0.71** | 0.11 | 0.14 | 0.13 |
| **3** | There is nothing wrong with sex dolls that look like children | | **0.64** | 0.20 | 0.20 | 0.19 |
| **4** | Viewing a nude or sexual image of a person under 18 is a victimless crime if the person doesn't know that the image was taken. | | **0.63** | 0.04 | 0.06 | 0.06 |
| **5** | I would still be friends with someone who I knew looked at nude or sexual images of people under 18. | | **0.69** | 0.13 | 0.25 | 0.05 |
| **6** | Boys under 18 are sexually experimental and are not harmed when they interact sexually with an adult online. | | **0.75** | 0.14 | 0.16 | 0.18 |
| **7** | It’s OK to flirt with people under 18 online if you don’t intend to take it further. | | **0.75** | 0.13 | 0.18 | 0.09 |
| **8** | If a 14 or 15-year-old teenager is on a dating app and contacting adults, they are at least partly responsible if an adult has a sexual interaction with them. | | **0.53** | -0.24 | 0.14 | -0.38 |
| **9** | Online sexual contact with a person under 18 that does not involve actual physical sexual contact or force is unlikely to harm that person psychologically. | | **0.66** | 0.02 | 0.14 | 0.02 |
| **10** | People under 18 on webcams usually come from poor backgrounds and providing them with money for sexual or nude services is helpful. | | **0.62** | 0.00 | 0.20 | 0.04 |
| **11** | People under 18 can make their own decisions about how much of their bodies they display on webcam. | | **0.69** | 0.01 | 0.18 | -0.03 |
| **12** | People under 18 who offer nude or sexual activity on livestream are exploring their sexuality and should not be censored. | | **0.73** | 0.16 | 0.22 | 0.08 |
| **13** | I would still be friends with someone who I knew had webcammed or livestreamed sexually with a person under 18. | | **0.69** | 0.20 | 0.30 | 0.06 |
| **14** | Viewing sexual images or videos of children is bad only because society says it is. | | **0.56** | 0.20 | 0.36 | 0.10 |
| **COMPONENT 2: Normalisation/blame diffusion** | | |  |  |  |  |
| **15** | Drawn, cartoon or computer-generated sexual imagery of children is not wrong (*reverse coded*). | | 0.11 | **0.73** | 0.03 | -0.03 |
| **16** | People under 18 cannot consent to online sexual interactions with adults (*reverse coded*). | | 0.06 | **0.68** | -0.01 | -0.17 |
| **17** | I would not be friends with someone who I knew had sexually interacted online with a person under 18 (*reverse coded*). | | 0.19 | **0.74** | 0.01 | -0.06 |
| **18** | It is always wrong to pay to view sexual activity with a child on a webcam, even if the child comes from a poor family and their parents need the money (*reverse coded*). | | 0.11 | **0.80** | -0.05 | 0.00 |
| **19** | If someone looks at online sexual images of people under 18 while under the influence of drugs and alcohol, they are still responsible for their actions (*reverse coded*) | | 0.10 | **0.78** | -0.05 | 0.04 |
| **COMPONENT 3: Restrictive stereotypes** | | |  |  |  |  |
| **20** | Sometimes people look at sexual images or videos of children because they are bored with normal adult pornography. | | 0.44 | 0.00 | **0.76** | -0.01 |
| **21** | Sometimes people look at sexual images or videos of children because they are very stressed. | | 0.36 | 0.09 | **0.77** | 0.04 |
| **22** | Some people look at sexual images or videos of children online to prevent themselves from sexually abusing children offline. | | 0.36 | 0.01 | **0.74** | 0.09 |
| **23** | Some people look at sexual images or videos of children because they were abused when they were children. | | 0.13 | -0.30 | **0.61** | 0.06 |
| **COMPONENT 4: Uncategorised** | | |  |  |  |  |
| **24** | Girls under 18 who share images of themselves nude or in revealing clothing are not to be blamed if an adult responds to them in a sexual way. | | 0.23 | -0.13 | 0.06 | **0.79** |
| **25** | People under 18 who act in sexual ways online are not to blame if an adult responds to them in a sexual way. | | 0.22 | -0.16 | 0.11 | **0.79** |
| Rotation Sums of Squared Loadings | | Eigenvalue | 6.77 | 3.21 | 2.67 | 1.59 |
|  |  | Percentage of variance | 27.09% | 12.84% | 10.70% | 6.34% |
| KMO = 0.943, Bartlett’s Test of Sphericity *x*^2^=54,946.67 (df=300), *p*<.001. | | | | | | |
